# Inhibition of Arenavirus Entry and Replication by the Cell-Intrinsic Restriction Factor ZMPSTE24 Is Enhanced by IFITM Antiviral Activity

**DOI:** 10.3389/fmicb.2022.840885

**Published:** 2022-02-18

**Authors:** Robert J. Stott-Marshall, Toshana L. Foster

**Affiliations:** Faculty of Medicine and Health Sciences, School of Veterinary Medicine and Science, Wolfson Centre for Global Virus Research, University of Nottingham, Loughborough, United Kingdom

**Keywords:** arenavirus, Lassa virus, ZMPSTE24, interferon-induced transmembrane proteins (IFITMs), cell-intrinsic factors, innate immunity

## Abstract

In the absence of effective vaccines and treatments, annual outbreaks of severe human haemorrhagic fever caused by arenaviruses, such as Lassa virus, continue to pose a significant human health threat. Understanding the balance of cellular factors that inhibit or promote arenavirus infection may have important implications for the development of effective antiviral strategies. Here, we identified the cell-intrinsic zinc transmembrane metalloprotease, ZMPSTE24, as a restriction factor against arenaviruses. Notably, CRISPR-Cas9-mediated knockout of ZMPSTE24 in human alveolar epithelial A549 cells increased arenavirus glycoprotein-mediated viral entry in pseudoparticle assays and live virus infection models. As a barrier to viral entry and replication, ZMPSTE24 may act as a downstream effector of interferon-induced transmembrane protein (IFITM) antiviral function; though through a yet poorly understood mechanism. Overexpression of IFITM1, IFITM2, and IFITM3 proteins did not restrict the entry of pseudoparticles carrying arenavirus envelope glycoproteins and live virus infection. Furthermore, gain-of-function studies revealed that IFITMs augment the antiviral activity of ZMPSTE24 against arenaviruses, suggesting a cooperative effect of viral restriction. We show that ZMPSTE24 and IFITMs affect the kinetics of cellular endocytosis, suggesting that perturbation of membrane structure and stability is likely the mechanism of ZMPSTE24-mediated restriction and cooperative ZMPSTE24-IFITM antiviral activity. Collectively, our findings define the role of ZMPSTE24 host restriction activity in the early stages of arenavirus infection. Moreover, we provide insight into the importance of cellular membrane integrity for productive fusion of arenaviruses and highlight a novel avenue for therapeutic development.

## Introduction

Viral haemorrhagic fever (VHF)-causing mammarenaviruses pose significant threats to human health, particularly in endemic regions of Western Africa; most are associated with high case fatality rates due to a lack of approved vaccines and effective countermeasures (Wolff et al., 2016; Whitmer et al., 2018; Kofman et al., 2019; Overbosch et al., 2020). These zoonotic enveloped RNA viruses possess single-stranded bi-segmented genomes and are members of the family *Arenaviridae* (McLay et al., 2014). The clinically significant, prototypic arenavirus lymphocytic choriomeningitis virus (LCMV), the most prevalent arenavirus Lassa (LASV), the pathogenic Lujo virus (LUJV), and the non-pathogenic Mopeia virus (MOPV), though phylogenetically closely related to LASV, are Old World arenaviruses that are mostly endemic in Western Africa. Junín (JUNV), Machupo (MACV) and Chapare (CHAPV) are New World arenaviruses that cause human viral hemorrhagic fevers endemic to South America (Bowen et al., 1996; Clegg, 2002). Seasonal outbreaks of LASV, the causative agent of Lassa Fever (LF) in several African countries, results in 100,000–300,000 infection cases annually and associated case fatality rates as high as 30% (Akpede et al., 2018; Asogun et al., 2019; NCDC, 2021). Recent expansion of LASV outside endemic regions has further emphasised the risk to public health and highlighted the urgency for effective vaccines and antiviral drugs. Therefore, unravelling key arenavirus-host interactions is critical toward this effort (Wolff et al., 2016; Whitmer et al., 2018; Kofman et al., 2019; Overbosch et al., 2020).

Virus entry into host cells is a key step of the virus lifecycle and is a determinant of virus replication, assembly, disease pathogenesis and virus emergence across host species. The arenavirus glycoprotein spike complex GP, consisting of subunits GP1, GP2 and the stable signal peptide (SSP) mediates cell attachment through interaction primarily with α-dystroglycan or transferrin 1 receptors at the plasma membrane for OW and NW viruses, respectively. Co-receptors including lysosome-associated membrane protein 1 (LAMP1) and the tetraspanin CD63 mediate fusion with cellular membranes following low-pH induced conformational changes in the GP structure (Di Simone and Buchmeier, 1995; Rojek et al., 2008; Abraham et al., 2009; Jae et al., 2014; Li et al., 2016; Raaben et al., 2017; Bulow et al., 2020).

Cell-intrinsic antiviral immunity plays a pivotal role in limiting this fusion step for a plethora of enveloped and non-enveloped viruses. This antiviral response involves constitutively expressed intrinsic immune proteins that act as the first line of defence against viral infection. The intrinsic innate response is enhanced through the activation of interferon (IFN)-stimulated genes (ISGs), following virus sensing by host-encoded pattern recognition receptors (PRRs), that further impose a cellular antiviral state (Bieniasz, 2004; Foster et al., 2016). These constitutive and induced factors inhibit at all steps of the virus life cycle, including the entry and fusion steps into cells (Foster et al., 2016; Shi et al., 2017, 2021).

Several intrinsic immune factors, also known as restriction factors, inhibit the infection of diverse enveloped viruses (Chemudupati et al., 2019; Stott et al., 2020). Amongst these factors are the human interferon-induced transmembrane protein (IFITM) family and the zinc metalloprotease, ZMPSTE24. As part of the robust IFN-mediated innate immune response to viral infection, three IFITMs display broad-range antiviral activity against enveloped viruses, including HIV-1, influenza virus and SARS-COV-2 (Brass et al., 2009; Everitt et al., 2013; Mudhasani et al., 2013; Desai et al., 2014; Suddala et al., 2019; Winstone et al., 2021). IFITM1 localises predominantly to the plasma membrane, whilst IFITMs 2 and 3 localise to early and late endosomal and lysosomal membranes (Weston et al., 2014; Foster et al., 2016). Current mechanisms of virus restriction are not clearly understood but existing explanations suggest that IFITMs inhibit viral fusion through a proximity-based mechanism in which they inhibit the formation of the fusion pore by trapping this process at the hemifusion stage (Desai et al., 2014; Suddala et al., 2019). IFITM homo-oligomerisation is thought to directly modify the structure, rigidity and curvature of target membranes, thus leading to a block in virus-host fusion (John et al., 2013; Desai et al., 2014). Indirect mechanisms have also been suggested, such as through alteration of membrane cholesterol composition or through endosomal association with other membrane proteins, such as ZMPSTE24 (Li et al., 2017).

ZMPSTE24, a seven-pass transmembrane protein, has recently been shown to be an intrinsic defence factor against IAV, Ebola, vaccinia and Zika viruses (Fu et al., 2017). ZMPSTE24 is constitutively expressed and localises to the inner nuclear membrane, and to multiple intracellular endocytic membrane compartments. Recruitment of ZMPSTE24 as a downstream effector of IFITM antiviral activity has been suggested to drive alterations in membrane properties that are less conducive to viral fusion (Li et al., 2017). However, the exact mechanism of this proposed restriction is currently unknown.

To date, there is limited information about how arenavirus infections may be antagonised by cell intrinsic factors (Radoshitzky et al., 2010; Chen et al., 2019; Stott et al., 2020), and the putative role of ZMPSTE24 in arenavirus biology has not yet been explored. In this study, we examined whether ZMPSTE24 is involved in arenavirus entry restriction. Using complementary arenavirus GP-pseudoparticle (GPpp) and live MOPV infection assays, we demonstrated that ZMPSTE24 restricts the entry and replication of arenaviruses. In agreement with previous reports, we found that arenavirus entry was resistant to IFITM protein overexpression (Huang et al., 2011; Suddala et al., 2019). We further show IFITM3 overexpression in the presence of ZMPSTE24 augmented restriction of arenavirus entry and replication and showed that ZMPSTE24 and IFITM3 can alter cellular endocytosis rates by impacting rigidity of cell membranes in an independent or cooperative manner. Collectively, our results provide strong support that arenaviruses utilise an endocytic pathway that is sensitive to ZMPSTE24 restriction and is enhanced upon recruitment of IFITM3.

## Materials and Methods

### Cell Lines and Expression Constructs

HEK 293T (ATCC), Vero (Vero; ATCC), A549 (ATCC) and A549 cells expressing ZMPSTE24 or the individual IFITM proteins were cultured in Dulbecco’s Modified Eagle Medium (DMEM), high glucose, GlutaMAX™ Supplement (Gibco) with 10% heat inactivated FBS (Gibco) and 200 μg/ml Gentamicin (Sigma) at 37°C, 5% CO_2_.

Expression plasmids encoding human ZMPSTE24 with and without a C-terminal FLAG-tag or HA-tag were PCR amplified and subcloned into the pQXCIP (Clontech) backbone using restriction sites *Age*I and *Bam*HI. Human IFITM1, IFITM2 and IFITM3 were cloned into the pLHCX retroviral vector (Clontech) using *Xho*I and *Not*I restriction sites.

Arenavirus glycoproteins for LCMV, LASV, MOPV, LUJV, JUNV, MACV, and CHAPV (accession numbers M22138, M15076, M33879, FJ952384, D10072, AY624355, and EU260463, respectively) were synthesised by GeneArt (ThermoFisher) and subcloned into the pI.18 expression vector using *Kpn*I and *Xho*I restriction sites.

A549 cells stably expressing the IFITMs 1, 2 or 3 (pLHCX) or ZMPSTE24 with or without a C-terminal FLAG tag (pQXCIP) or the relevant empty vector, were generated by vesicular stomatitis virus-G (VSV-G) pseudotyped retroviral transduction. Retroviral vectors were made by transfecting 293T cells with the pCMV-Gag-Pol murine leukaemia virus (MLV) packaging construct, the pLHCX or pQXCIP packaging vector of interest and pCMV VSV-G using 1 mg/ml PEI^®^-MAX (Polysciences). Stable A549 cells were generated by spinoculation with retroviral vectors and antibiotic selection. Expression of proteins was assessed by western blotting. When indicated, IFN1 (universal type 1 IFN, PBL Interferon Source) stimulation was performed using 1000 U/ml^–1^ for 4 h before immunoblotting.

CRISPR-Cas9 gRNA sequences to target human ZMPSTE24 (CACAACTAATGTGAACAGCC) and a non-targeting control (GGCCCTCTAGAAAAGTCTCG) were generated in pLentiCRISPR v2 and gRNA sequences to target human IFITMs 1, 2 and 3 (TTCTTCTCTCCTGTCAACAG) were generated in eSpCas9-LentiCRISPR v2 (Genscript). Viral stocks were generated in 293T cells by co-transfection with psPAX2 (Addgene), pMD2.G VSV-g and the eSpCas9-LentiCRISPRv2 construct targeting ZMPSTE24 or the IFITMs or a non-targeting control. A549 cells transduced with the pLentiCRISPR viruses were selected with antibiotics for 7–14 days. Efficiency of knockout was determined by western blot.

The NanoBiT split luciferase system (Promega) was used to assess interaction of IFITM3 and ZMPSTE24 as per manufacturer’s instructions. IFITM3 and ZMPSTE24 were fused to NanoBiT large (LgBiT) or small (SmBiT) subunits of NanoLuc luciferase at the N or C terminus by restriction digest cloning.

### Passage, Titration and Infection With Mopeia Virus

The UVE/MOPV/UNK/MZ/Mozambique 20410 strain of MOPV was obtained from European Virus Archive goes Global (EVAg) platform and mycoplasma-free stocks were propagated in Vero cells in DMEM supplemented with 2% FCS. The titre was determined by plaque immunoassay. Vero cells were infected with serial dilutions of MOPV and incubated in MEM with 2.5% low viscosity carboxymethylcellulose (Merck) for 4 days. Plaques were visualised using mouse monoclonal anti-Arenavirus (OW) rGPC, clone KL-AV-1B3 (BEI Resources, 1:500) followed by anti-mouse Alkaline Phosphatase secondary (Merck, 1:750) and addition of BCIP/NBT substrate. Virus titres were calculated as plaque forming units per mL (PFU/mL).

A549 cells were infected with MOPV at an MOI 0.01 and incubated at 37°C. For interferon treatment, cells were treated with 1000 U/ml^–1^ IFN1 for 4 h before infection. To assess the effect of amphotericin B, the relevant stable A549 cell lines were incubated with 1 μM amphotericin B (Sigma Aldrich) for 1 h at 37°C prior to and following infection with MOPV.

### Generation of Arenavirus GP Retroviral Pseudoparticles

Arenavirus GP-expressing pseudoparticles (GPpp) encoding GFP were produced by transfecting 293T cells with pCMV-MLV gag-pol, pCMV-MLV GFP encoding a CMV-GFP internal transcriptional unit and the pI.18 plasmid encoding the arenavirus GP of interest, at a ratio of 0.6:0.9:0.6 μg using 1 mg/ml PEI MAX^®^. GPpp supernatants were harvested through a 0.45 μm filter 48 h post transfection. GPpp supernatants were titrated on A549 cells by flow cytometry, performed using a BD FACSCanto II flow-cytometer (Becton Dickinson), collecting 10,000 events, and analysed using FlowJo software.

Cells were infected with arenavirus GP retroviral pseudoparticles, encoding GFP at an MOI of 0.3 in complete growth media and incubated at 37°C for 48 h. Infected cells were analysed by flow cytometry. Samples were gated on live cells for 10,000 events and analysed for expression of GFP. To test the effect of IFN1 on arenavirus GP mediated cell entry, cells were treated with IFN1 (universal type 1 IFN, PBL Interferon Source) for 4 h prior and throughout infection. To assess the effect of amphotericin B on the restriction of arenavirus entry by ZMPSTE24 and the co-operative action with IFITMs, relevant stable A549 cell lines were treated with 1 μM amphotericin B (Sigma Aldrich) for 1 h at 37°C prior to infection and following infection with arenavirus GP pseudoparticles.

### β-Lactamase-Vpr Assay

Briefly, β-lactamase-Vpr (BlaM-Vpr)-chimaera pseudotyped viruses were produced by co-transfection of 293T cells with a pNL4.3 R- E-, pCMV BlaM-Vpr, pAdvantage and plasmids encoding the GP of MOPV, LCMV or MLV. A549 cells control or ZMPSTE24 overexpressing cells were plated the day prior to infection. Cells were pretreated with 50 nM Bafilomycin A1 (BafA1) for 30 min and then mock infected or were incubated with 100 ng p24 BlaM-Vpr chimaera pseudotyped viruses for 3 h. Cells were washed once in CO_2_-independent media and loaded with CCF2-AM substrate (Invitrogen) containing development media (CO_2_-independent media containing 1.6 mM probenecid) for 2 h at room temperature. Cells were then washed twice in development media before a final 16 h incubation at room-temperature in development media. Cells were harvested in trypsin, washed and fixed in 4% PFA before analysis on the ID7000 Spectral Cell Analyser (Sony Technology).

### Immunoblotting

Cells were lysed in 2x reducing Laemmli buffer (Bio-Rad) at 100°C for 10 min. Samples were separated on 4–15% Mini-PROTEAN^®^ TGX Precast gels (Bio-Rad) and transferred onto 0.2 μm nitrocellulose membrane (Bio-Rad). Membranes were blocked in 5% milk in PBS with 0.1% Tween20 (PBS-T) prior to incubation with primary antibodies: mouse anti-IFITM1 (Proteintech, 60074-1-Ig, 1:5000), rabbit anti-IFITM2 (Proteintech, 12769-1-AP, 1:5000), rabbit anti-IFITM3 (Proteintech, 11714-1-AP, 1:5000), rabbit anti-ZMPSTE24 antibody (Abcam ab38450, 1:1000), mouse anti-FLAG (Sigma, F1804, 1:2000), mouse anti-HA (Abcam ab18181, 1:5000), mouse anti-HSP90 (Invitrogen, MA1-10372, 1:10,000). This was followed by horseradish peroxidase (HRP)-conjugated horse anti-mouse IgG (CST, 7076S, 1:5000) or goat anti-rabbit IgG (CST, 7074S, 1:5000) secondary antibodies and detection using SuperSignal™ West Pico PLUS Chemiluminescent Substrate (ThermoFisher).

### Immunoprecipitations

For the endogenous immunoprecipitation (IP), A549 cells were treated with 1000 U/ml^–1^ IFN1 for 24 h before lysis on ice for 20 min in 50 mM Tris-HCL pH 7.4, 150 mM NaCl, 1% IGEPAL^®^CA-630 (Sigma), complete protease inhibitors (Roche). For the co-IP, A549 cells pre-treated with 1000 U/ml^–1^ IFN1 and then were mock-transfected or transfected with 2 μg pQXCIP ZMPSTE24-FLAG. 48 h post transfection cells were lysed on ice as above. Lysed samples were centrifuged and supernatants were immunoprecipitated with 5 μg/ml mouse monoclonal anti-IFITM2/3 antibody (Proteintech, 66081-1-Ig) for 1.5 h at 4°C. Protein G agarose (ThermoFisher) was equilibrated in lysis buffer before adding to supernatants and incubated overnight at 4°C. Following extensive washes in lysis buffer, cell lysates and immunoprecipitates on beads were resuspended in 2x Laemmli buffer (Bio-Rad) and resolved by SDS-PAGE and western blot analysis.

### Immunofluorescence

A549 cells grown on coverslips were fixed with 4% paraformaldehyde (PFA) in PBS permeabilized with 0.2% Triton X-100 in PBS for 15 min at RT prior to incubation with 0.1 M glycine for 20 mins. Primary antibodies, diluted in 3% BSA, 0.1% Tween^®^20 in PBS, were incubated overnight at 4°C (rabbit anti-ZMPSTE24, Abcam ab38450, 1:200 or mouse monoclonal anti-HA, Abcam ab18181, 1:500 or rabbit anti-EEA1, CST 2411S, 1:100 or rabbit anti-Rab9A, CST 5118T, 1:200, or rabbit anti-LAMP1, Invitrogen 14-1079-80, 1:500). Coverslips were incubated for 1h at RT with appropriate secondary antibodies conjugated to Alexa Fluor 488 or 594 (Molecular Probes, ThermoFisher 1:500) diluted in 3% BSA, 0.1% Tween20 in PBS. Coverslips were mounted using Fluoroshield™ with DAPI (Merck). Images were acquired on a Zeiss LSM880 confocal laser scanning microscope or on a Leica DM5000 B widefield microscope. Pearson’s correlation coefficient was calculated using ImageJ software.

### NanoBiT Protein Interaction Assay

A NanoBiT protein:protein interaction assay (Promega) was used to assess the interaction between ZMPSTE24 and IFITM3. A549 cells were transiently co-transfected with 100 ng in total of an N- or C-terminal LgBiT and SmBiT tagged construct using Lipofectamine™ 3000 Transfection Reagent (ThermoFisher). All possible combinations of the N-terminal and C-terminal-tagged split luciferase protein pairs were tested. All LgBiT constructs were co-transfected with the HaloTag-SmBiT (negative control) construct against which relative luminescence was measured. Luminescence was measured after 48 h using the Nano-Glo Live Cell Assay System (Promega) according to manufacturer’s instructions.

### RT-qPCR Analysis

Nucleoprotein (NP) and RNA-dependent RNA polymerase (L) RNA levels were determined in cells infected with MOPV at specified time points. Total RNA was isolated from infected cells using QIAGEN RNeasy Plus Mini Kit (Qiagen) and cDNA was reverse transcribed using the Applied Biosystems*^rmTM^* High-Capacity cDNA Reverse Transcription Kit. For each qPCR reaction, 20 ng of cDNA was used with the Applied Biosystems™ PowerUp™ SYBR™ Green Master Mix. Primers used were as follows: GAPDH forward (5′-ACATCGCTCAGACACCATG-3′); GAPDH reverse (5′-TGTAGTTGAGGTCAATGAAGGG-3′); β-actin forward (5′-CACCAACTGGGACGACAT-3′); β-actin reverse (5′-ACAGCCTGGATAGCAACG-3′); MOPV L forward (5′-ATCTCCTCATGCAGCCACAC-3′); MOPV L reverse (5′-GGACTGTTGGAGAGTTGCGA-3′); MOPV NP forward (5′-CCCTGGCATGTCAAGACCAT-3′); MOPV NP reverse (5′-CCCTGTGGAAGTTGCGATCT-3′). Primer specificity was confirmed by melt curve analysis. Relative fold expression of target genes was normalised to reference genes GAPDH and β-actin by the ^Δ^ ^Δ^ Ct method.

### Membrane Fusion Assay

Mopeia virus particles were labelled with the self-quenching dye SP-DiOC18 (Invitrogen). SP-DiOC18 was added to 1 ml of MOPV virus stock at 1 × 10^6^ PFU/ml at a final concentration of 0.2 μM. The mixture was protected from light and incubated with gentle rolling for 1 h at room temperature. For the heat inactivated control virus was incubated at 75°C for 30 min and cooled to room temperature before labelling. The labelled virus preparations were passed through a 0.45 μM filter before use and diluted in serum-free DMEM for infection of A549 monolayers at an MOI of 5. Cells were incubated with labelled virus at 4°C for 15 min and then incubated for 1.5 h at 37°C after infection. Cells were then harvested with trypsin, fixed with 4% PFA for 10 min and then analysed by flow cytometry on the FACSCanto II (BD Biosciences) using the 488 nm laser for excitation.

### FM2™-10 Incorporation Assay

The detection of FM™2-10 (N-(3-Triethylammoniumpropyl)-4-(4-(Diethylamino)styryl) Pyridinium Dibromide, ThermoFisher) fluorescence intensity as a function of time was used as a measure of endocytosis. A549 cells stably expressing ZMPSTE24, or IFITM3 or ZMPSTE24 and IFITM3 in combination or pQXCIP empty vector were washed and resuspended in PBS. A 2 μM stock solution of FM™2-10 was prepared in PBS before adding cells to a final concentration of 200 nM and incubating for 5, 10, 30, and 60 min. Changes in FM™2-10 fluorescence intensity over time were detected by flow cytometry for each cell sample.

### Statistical Analysis

Statistical analyses were carried out using IBM SPSS or GraphPad Prism v9.0.2. Data was subjected to normality testing followed by ANOVA with LSD *post hoc* testing or independent samples *t*-tests. Statistical significance is denoted by asterisks as follows: ^∗^ *p* < 0.05, ^∗∗^ *p* < 0.001, ^∗∗∗^ *p* < 0.0001.

## Results

### ZMPSTE24 Impairs Arenavirus GPpp Infection

**FIGURE 1:**
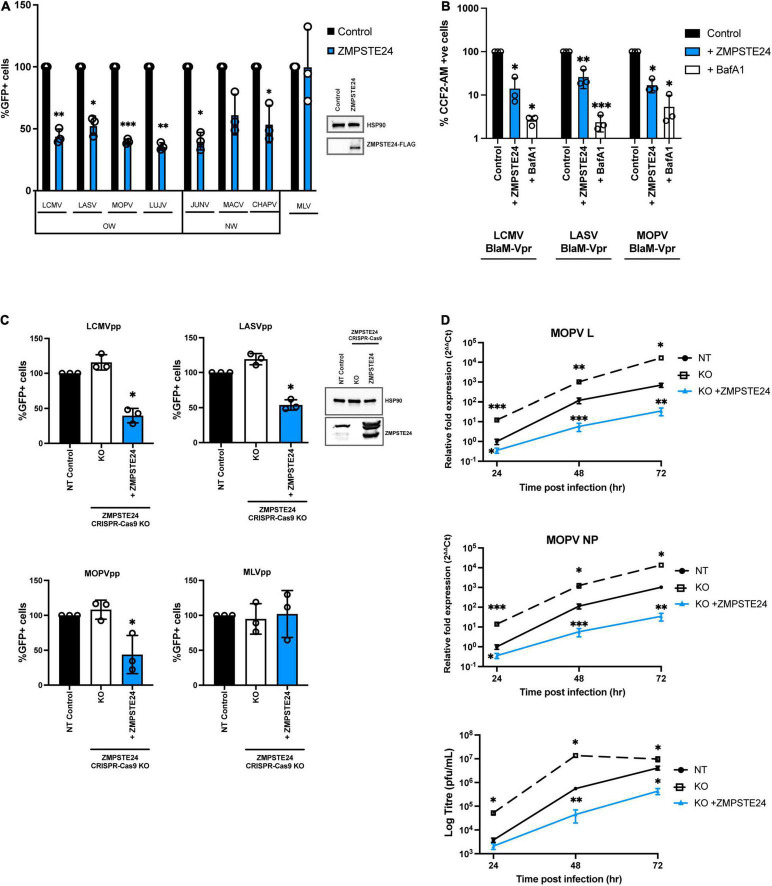
Arenavirus entry and replication is inhibited by ZMPSTE24. **(A)** GFP-containing GP pseudoparticles (GPpp) from Old world (OW) and New world (NW) arenaviruses or an amphitropic murine leukaemia virus (MLV) control were used to infect A549 cells stably expressing ZMPSTE24-FLAG or an empty vector control (inset). GPpp entry was measured by flow cytometry and expressed as %GFP + cells relative to controls. **(B)** Control or ZMPSTE24 expressing A549 cells were mock-infected or infected for 3 h with LCMV, LASV or MOPV GP-pseudotyped viruses carrying the BlaM-Vpr fusion protein at 100 ng p24. Cells were loaded with fluorescent substrate of beta-lactamase, CCF2-AM and virus entry into the cytoplasm was assessed by cleaved CCF2-AM fluorescence at 450 nm by flow cytometry. **(C)** A549 cells stably expressing either non-targeting (NT) control, ZMPSTE24 CRISPR-Cas9 knockout (KO) or ZMPSTE24 KO with ZMPSTE24-FLAG overexpression, were infected with arenavirus GPpp or MLVpp control and infection was measured by flow cytometry. **(D)** Cells in panel C were infected with live MOPV (MOI = 0.01). Relative fold expression of MOPV L or NP genes were measured by RT-qPCR from extracted RNA and infectious virus production was measured by plaque assay of cell supernatants at 24, 48, and 72 h post infection. Data are shown as mean ± SE (standard error) of *n* = 3 independent experiments. Significance is indicated as *p*-values: *** *p* < 0.0001, ** *p* < 0.001, *p < 0.05.

To examine the role of ZMPSTE24 restriction during arenavirus entry, we used arenavirus GP–pseudoparticles (GPpp) generated from a panel of OW (LCMV, LASV, LUJV, MOPV) and NW (JUNV, MACV, CHAPV) mammarenavirus representatives. Specifically, murine leukaemia virus (MLV) packaging a green fluorescent protein (GFP) reporter was pseudotyped with different arenavirus GP proteins. We also included an amphotropic MLV GPpp as a control. These pseudoparticles are replication-incompetent, recombinant retroviral particles that allow GP-mediated entry phenotypes to be measured (Carnell et al., 2015). Using flow cytometry analysis, we first investigated the infectivity of these arenavirus GPpp in A549 human lung epithelial cells stably expressing FLAG-tagged ZMPSTE24 compared to vector control cells. Entry of both OW and NW arenavirus GPpp was inhibited in ZMPSTE24 expressing cells compared to the control. For the MLV pseudoparticles, entry occurs by macropinocytosis and we observed little effect of ZMPSTE24 expression on MLV infection (Figure 1A and Supplementary Figure 1A) (Rasmussen and Vilhardt, 2015). Concordantly, using BlaM-Vpr-chimaera particles pseudotyped with glycoproteins from LCMV, LASV, MOPV, and MLV that would induce the cleavage of CCF2-AM dye upon cytosolic entry, we independently demonstrated that expression of ZMPSTE24 decreased the fusogenicity of arenavirus GP-pseudotyped particles (Figure 1B and Supplementary Figure 1B). We then mutated histidine residue 335 within the essential, conserved HEXXH zinc metalloprotease catalytic motif to examine the importance of ZMPSTE24 metalloprotease activity in the restriction of arenavirus GPpp entry. The H335A mutant also displayed comparable restriction of LCMVpp and LASVpp infection to wild-type ZMPSTE24 in A549 cells when compared with vector only controls, albeit to a slightly lesser extent (Supplementary Figure 1C). To complement these studies, we depleted the expression of ZMPSTE24 in A549 cells using CRISPR-Cas9 lentiviral vectors. We first observed a modest increase in GPpp entry in ZMPSTE24 KO cells by most OW and NW arenaviruses (Figure 1C and Supplementary Figure 1D). Upon stable overexpression by retroviral transduction of ZMPSTE24-FLAG, in these ZMPSTE24 KO cells, we observed a marked decrease in LCMVpp, LASVpp, and MOPVpp infection, but not that of MLVpp (Figure 1C). Therefore, the combined data suggest that ZMPSTE24 impedes arenavirus entry and fusion but that this viral restriction activity is largely independent of its protease function.

To examine the activity of ZMPSTE24 in the context of a live virus infection, we assessed intracellular MOPV L and NP gene expression and infectious MOPV production over 72 h in the A549 ZMPSTE24 KO cells and KO cells overexpressing ZMPSTE24-FLAG, as described above. Depletion of ZMPSTE24 in these cells resulted in significantly higher levels of MOPV genome and infectious virus production throughout the course of infection (Figure 1D and Supplementary Figure 1E). Expression of ZMPSTE24-FLAG in these KO cells dramatically decreased intracellular virus genome and infectious virus production (Figure 1D) demonstrating a significant antiviral role of ZMPSTE24 during live arenavirus infection.

### Arenavirus GPpp Entry Is Insensitive to IFITM Protein Overexpression

**FIGURE 2:**
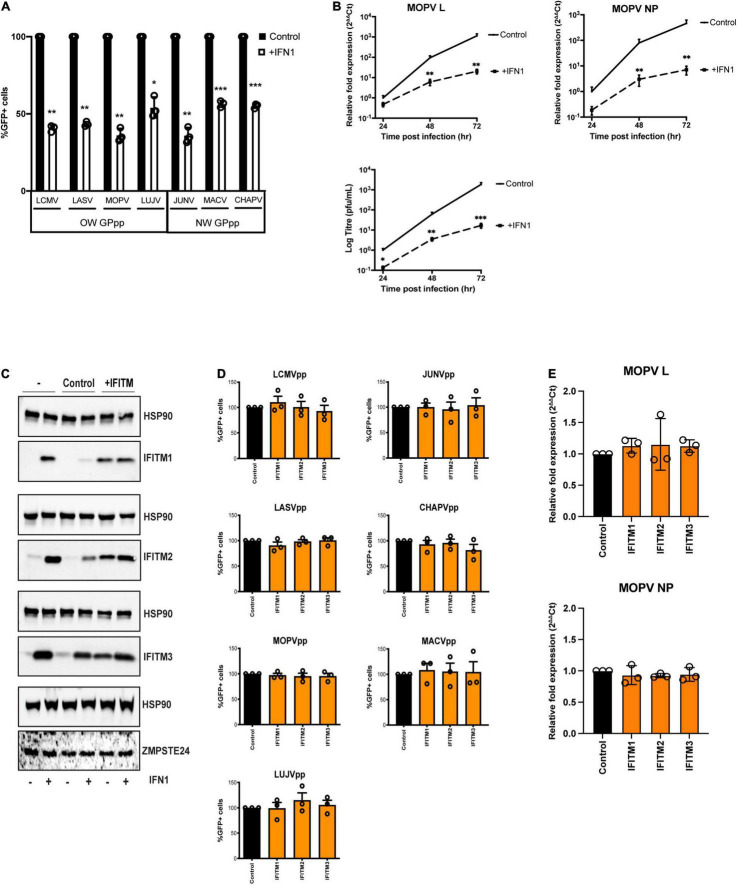
Arenavirus GPpp entry and MOPV replication is inhibited by IFN but is insensitive to IFITM overexpression. **(A)** A549 cells were infected with GFP-containing arenavirus GPpp in the presence or absence of type 1 interferon (IFN1) and infection was measured by flow cytometry. **(B)** A549 cells were infected with live MOPV (MOI = 0.01) in the presence or absence of IFN1. MOPV L or NP gene expression was determined by RT-qPCR of extracted RNA and infectious virus in cell supernatants was measured by plaque assay at indicated times post infection. **(C)** A549 cells were transduced with empty vector control pLHCX, IFITM1, IFITM2 or IFITM3. Expression of IFITMs and ZMPSTE24 was measured in the presence or absence of 1000 U/mL IFN1 by western blot analysis. HSP90 served as a loading control. A549 cells stably transduced with IFITMs were infected with arenavirus GPpp and% infectivity measured by flow cytometry **(D)** or were infected with live MOPV (MOI = 0.01) for 72 h and MOPV L or NP gene expression was measured by RT-qPCR of extracted RNA **(E)**. Data are shown as mean ± SE (standard error) of *n* = 3 independent experiments. Significance is indicated as *p*-values *** *p* < 0.0001, ** *p* < 0.001, *p < 0.05.

It has been suggested that ZMPSTE24 is recruited to endocytic compartments by IFITM proteins, thereby blocking the endocytic entry of enveloped viruses (Fu et al., 2017; Li et al., 2017). We aimed to examine the role that IFITMs play in the restriction activity against arenaviruses. We first assessed the antiviral effects of exogenous IFN1 on the early stages of arenavirus infection in A549 cells. Single-round infectivity of GPpp across OW and NW strains was markedly reduced in cells incubated with 1000 U/ml universal IFN1 (Figure 2A). We then treated A549 cells with universal IFN1 and challenged them with live MOPV, measuring over 72 h MOPV gene expression levels and infectious MOPV production. We found that MOPV infection was highly sensitive to IFN1 (Figure 2B and Supplementary Figure 2A). These data infer the likely contribution of interferon-induced factors that can limit MOPV infection, including those involved at the early entry stage. It has previously been reported that arenaviruses are not susceptible to restriction by overexpressed IFITM proteins in cells (Huang et al., 2011; Suddala et al., 2019). To validate this, we generated A549 cells stably expressing individual human IFITM1, IFITM2 and IFITM3 proteins at levels similar in magnitude to that induced by IFN1 treatment (Figure 2C). By contrast, the expression of endogenous ZMPSTE24 was not upregulated following treatment with IFN1. We analysed the infectivity of different arenavirus GPpp in these cells and found that all strains tested appeared resistant to IFITM overexpression (Figure 2D). We also infected IFITM-expressing cells with live MOPV for 72 h and showed that IFITM overexpression had no effect on MOPV gene expression (Figure 2E). These data show that arenavirus entry and replication is not restricted by IFITMs alone, in agreement with previous studies (Huang et al., 2011; Suddala et al., 2019).

### ZMPSTE24 and IFITM Proteins Interact *via* Their C-Termini

**FIGURE 3:**
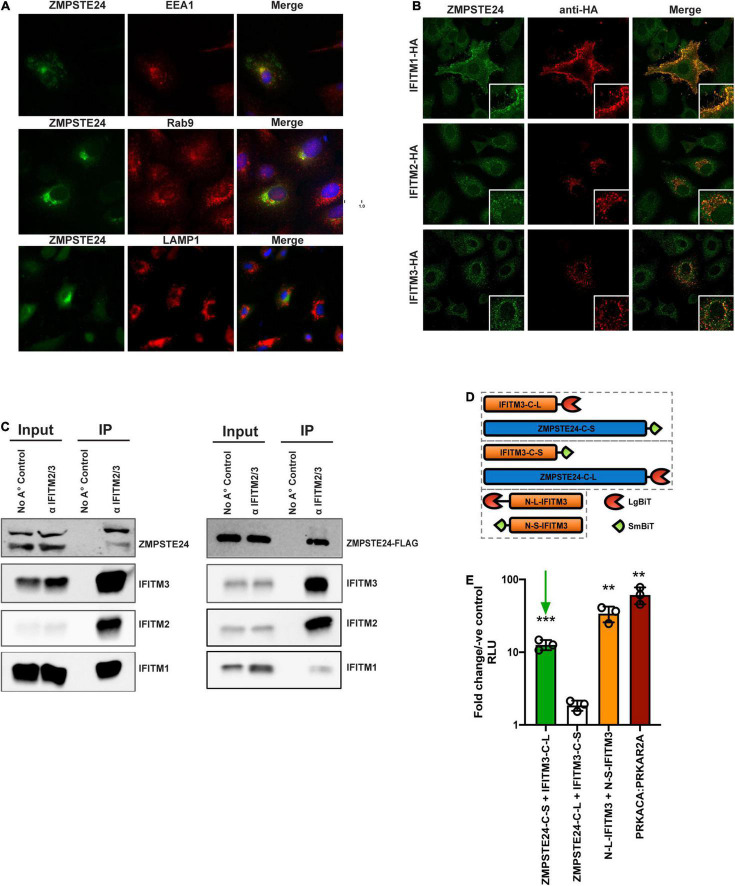
ZMPSTE24 colocalises with IFITM proteins and interacts *via* a C-terminal interaction with IFITM3. **(A)** ZMPSTE24 is localised to early and late endosomal compartments. ZMPSTE24-HA was transiently expressed in A549 cells which were then probed with antibodies against HA, early endosomal (EEA1) or late endosomal (Rab9) markers, or LAMP1. Nuclei were counterstained with DAPI. Panels are of representative images. Pearson’s correlation coefficient of ZMPSTE24 with the early and late endosomal compartment markers was calculated by measuring cells of interest using ImageJ software. **(B)** A549 cells transiently expressing HA-tagged IFITM proteins 1, 2, and 3 were probed with antibodies against endogenous ZMPSTE24 and HA and imaged by confocal microscopy. Panels are of representative images. Pearson’s correlation coefficient of ZMPSTE24 with individual IFITM proteins was calculated by measuring cells of interest using ImageJ software. **(C)** For the endogenous IP, A549 cells were pre-treated with IFN prior to cell lysis. For the co-IP, IFN pre-treated A549 cells were mock-transfected or transfected with ZMPSTE24-FLAG. Cell lysates were immunoprecipitated with anti-IFITM2/3 monoclonal antibody prior to resolving by SDS-PAGE and analysis by western blotting for endogenous ZMPSTE24 or ZMPSTE24-FLAG along with IFITMs 1, 2, and 3. **(D)** Schematic illustration of NanoBiT construct expression. IFITM3 or ZMPSTE24 were fused to large (LgBiT) or small (SmBiT) subunits of NanoLuc luciferase at N or C terminals and co-transfected in pairs (hatched boxes) into HEK293T cells. **(E)** Luminescence produced by interaction of co-expressed NanoBiT pairs was measured in live cells and analysed relative to a control consisting of the target-LgBiT co-transfected with a HaloTag-SmBiT control. LgBiT-PRKACA and SmBiT-PRKAR2A were co-transfected as a positive control. Green arrow indicates interaction between ZMPSTE24-C-SmBiT and IFITM3-C-LgBiT. Data are shown as mean ± SE (standard error) of *n* = 3 independent experiments. Significance is indicated as *p*-values *** *p* < 0.0001, ** *p* < 0.001.

To address the effects of IFITMs on ZMPSTE24 activity, we first assessed their comparative localisation. Microscopy studies revealed that overexpressed HA-tagged ZMPSTE24 possesses a cytoplasmic distribution and predominantly localises to endosomal compartments in A549 cells. Further, we found that ZMPSTE24 co-localises with the early endosome marker, EEA1 (Pearson’s coefficient 0.634) and to a lesser extent with the late endosome marker Rab9 (Pearson’s coefficient 0.496) but has very low localisation with the lysosome marker LAMP1 (Pearson’s coefficient 0.166; Figure 3A). The localisation of IFITM proteins is thought to define the spectrum of viruses that these antiviral factors restrict (Shi et al., 2017). IFITM1 is mostly localised to the plasma membrane, whilst IFITMs 2 and 3, like ZMPSTE24, are localised to endosomal compartments due to the presence of a conserved endocytic localisation motif (Foster et al., 2016; Shi et al., 2017). We demonstrated by confocal microscopy imaging that endogenous ZMPSTE24 co-localised with HA-tagged IFITM1, IFITM2 and IFITM3 proteins when overexpressed in A549 cells (Pearson’s coefficients 0.769, 0.580, and 0.464, respectively; Figure 3B). In the presence of IFITM1, ZMPSTE24 redistributed to the plasma membrane and IFITM1 was also found to have a disperse intracellular punctate distribution that overlapped with ZMPSTE24. This observation implies a cooperative function or interaction of the two proteins (Figure 3B). Considering these observations, we next corroborated that IFITMs and ZMPSTE24 interact (Fu et al., 2017; Li et al., 2017). A549 cells were either pre-treated with IFN1 for 24 h and immediately subjected to immunoprecipitation or transfected with C-terminally FLAG-tagged ZMPSTE24. IFITM proteins have the propensity to homo- and heterooligomerise, therefore, we captured the proteins on beads coated with anti-IFITM2/3 antibody and the assays analysed by western blot (John et al., 2013). We found that endogenous ZMPSTE24 or ZMPSTE24-FLAG bound to the endogenous IFITM1, 2 and 3 proteins, further supporting that ZMPSTE24 interacts with individual IFITMs and could interact oligomers of these proteins (Figure 3C). To complement this, we assessed the interaction between ZMPSTE24 and IFITM3, in live cells using a NanoLuc Binary Technology (NanoBiT)-based assay (Figure 3D). NanoLuc Luciferase is split into two complementary segments, 18kDa Large BiT (LgBiT) and 1.3kDa Small BiT (SmBiT); these possess low intrinsic affinity for each other. However, a bright luminescent signal is restored upon interaction of the binding partners to which they are fused. We engineered ZMPSTE24 and IFITM3 constructs tagged at either the N or C terminus with SmBiT and LgBiT fragments (Figure 3D). We transiently transfected HEK293T cells with these combinations of SmBiT/LgBiT ZMPSTE24 and IFITM3 constructs to screen for conformational interactions by detection of a luminescence signal. As IFITMs are known to oligomerize *via* the N-terminus, we included a co-transfection of N-terminal LgBiT279 IFITM3 (N-L-IFITM3) and N-terminal SmBiT-IFITM3 (N-S-IFITM3) as indication of a positive interaction. Luminescence signal was compared to the manufacturer’s PRKACA:PRKAR2A positive control pair and the negative control of the corresponding SmBiT partner fused with a HaloTag. Co-transfection of N-L-IFITM3 and N-S-IFITM3 produced a robust signal when normalised to the negative control, indicating the oligomerisation of IFITM proteins (Figure 3E). We found that C-terminal tagged ZMPSTE24 (ZMPSTE24-C-S) and IFITM3 (IFITM3-C-L) in combination produced a luminescent signal approximately 12-fold higher than that of the negative control pairs and approaching the signal of the N-L-IFITM3/N-S-IFITM3 combination (Figure 3E), suggestive of a C-terminal interaction between ZMPSTE24 and IFITM3.

### IFITM Proteins Contribute to the Antiviral Restriction of Arenavirus Entry by ZMPSTE24

**FIGURE 4:**
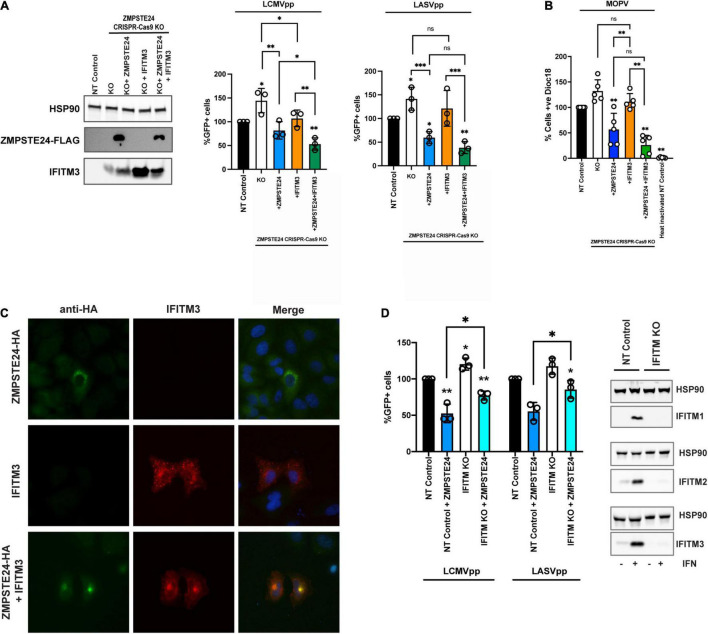
ZMPSTE24 and IFITM3 co-operatively restrict arenavirus infection. **(A)** A549 CRISPR-Cas9 stable knock out cell lines were generated for non-targeting (NT) control or ZMPSTE24 and then transduced for stable overexpression of ZMPSTE24-FLAG or IFITM3 or both. Stable expression was confirmed by western blot cells were then infected with LCMVpp or LASVpp and% infection was measured by flow cytometry analysis of GFP expression. **(B)** Cells from panel A were infected with SP-DiOC-18 labelled MOPV for 1.5 h. SP-DiOC18 positive cells were measured by flow cytometry. **(C)** A549 cells were transiently transfected with HA-tagged ZMPSTE24 (green) and IFITM3 (red) and co-localisation was assessed by immunofluorescence microscopy. Nuclei were counterstained with DAPI. **(D)** A549 CRISPR-Cas9 NT control or IFITM KO cells were generated, and expression was assessed by western blot. Stable cells were then transduced express ZMPSTE24-FLAG and infected with LCMVpp or LASVpp. Infection was measured as %GFP positive by flow cytometry. Data are shown as mean ± SE (standard error) of *n* = 3 independent experiments. Significance is indicated as *p*-values *** *p* < 0.0001, ** *p* < 0.001, *p < 0.05.

We aimed to ascertain if IFITMs play a role in the ZMPSTE24-mediated restriction of arenavirus entry. We assessed the infectivity of LCMVpp and LASVpp in A549 cells with CRISPR-Cas9 KO of endogenous ZMPSTE24 and overexpressing either ZMPSTE24-FLAG or IFITM3, or both proteins together by retroviral transduction (Figure 4A). As anticipated, ZMPSTE24 knockout increased LCMVpp and LASVpp infection but this was abrogated in the presence of ZMPSTE24-FLAG. IFITM3 overexpression alone had little effect on GPpp infection but in combination with ZMPSTE24-FLAG, we observed a significant enhancement in the restriction of LCMVpp and LASVpp infection (Figure 4A and Supplementary Figure 2B). Upon labelling MOPV particles with the self-quenching dye SP-DiOC18 for which fluorescence dequenching occurs upon viral membrane fusion with endosome, we measured a significant decrease in fusion events in the presence of ZMPSTE24 compared to control and ZMPSTE24 KO cells (Figure 4B). IFITM3 expression alone had little effect on fusion, but co-expression with ZMPSTE24 enhanced the restriction of viral-endosome fusion events in these cells (Figure 4B).

Interestingly, when we examined A549 cells overexpressing ZMPSTE24 and IFITM3 in combination by microscopy, we found that the ectopic expression of both ZMPSTE24 and IFITM3 resulted in a redistribution of the two proteins from their disparate endo-cytoplasmic localisation to a distinct endosomal, pre-nuclear co-localisation (Figure 4C). This sub-cellular localisation of ectopic ZMPSTE24 and IFITM3 could represent a compartment where arenavirus and host membrane fusion occurs and warrants further investigation into its function. Thus, we speculate that this redistribution of IFITM3 likely influences the observed enhancement in ZMPSTE24 restriction of arenavirus entry.

We next generated A549 lentiviral non-targeting (NT) control and CRISPR-Cas9 IFITM knockout cells and stably expressed ZMPSTE24-FLAG in these cells. Compared to NT control cells, we observed enhanced LCMVpp and LASVpp infection in IFITM KO cells (Figure 4D). Overexpression of ZMPSTE24 in NT control cells abrogated LCMVpp and LASVpp infection but in the absence of IFITM expression, the sensitivity of arenavirus entry to ZMPSTE24 restriction was decreased (Figure 4D and Supplementary Figure 2C).

Taken together, our data show that ZMPSTE24 interacts with IFITM proteins and importantly suggest a cooperative impairment of arenavirus entry in which ZMPSTE24 appears to co-opt IFITM3 to facilitate restriction of arenavirus entry.

### ZMPSTE24 Restriction of Arenavirus Infection Is Mediated Through Modulation of Membrane Integrity

**FIGURE 5:**
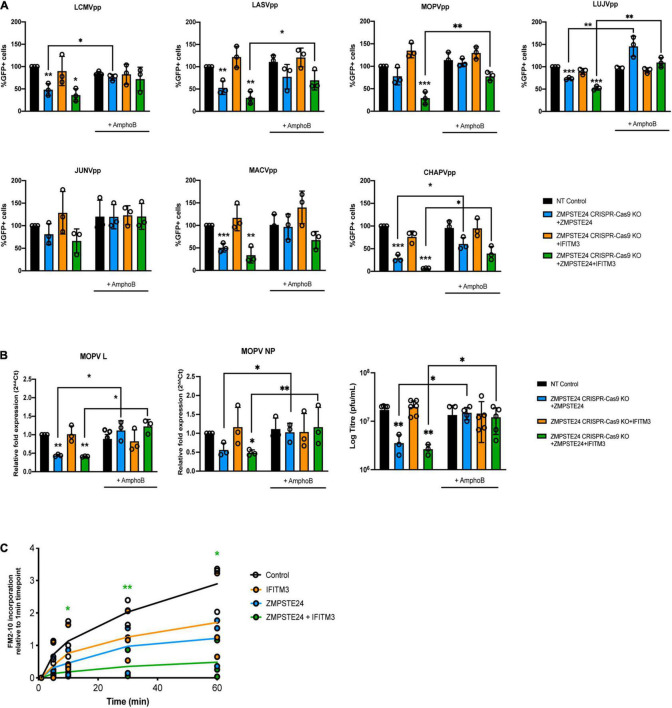
ZMPSTE24 and IFITM3 modulate membrane fluidity to restrict arenaviruses. A549 CRISPR-Cas9 non-targeting (NT) or ZMPSTE24 knockout (KO) cells were transduced for stable expression of ZMPSTE24-FLAG, IFITM3 or both. Cells were incubated with 1 μM Amphotericin B (AmphoB) for 1 h prior to infection with **(A)** arenavirus GPpp for 48 h or with **(B)** MOPV (MOI = 0.01) for 72 h. GPpp infectivity was measured as %GFP positive by flow cytometry. MOPV L and NP gene expression was measured by RT-qPCR from extracted RNA while infectious virus production was measured by plaque assay from cell supernatants. **(C)** A549 cells stably expressing ZMPSTE24-FLAG, IFITM3 or both were incubated with 200 nM FM™2-10 for indicated time points and FM™2-10 incorporation was measured as fluorescence intensity of incorporated membrane probe by flow cytometry. Data are shown as mean ± SE (standard error) of *n* = 3 independent experiments. Significance is indicated as *p*-values *** *p* < 0.0001, ** *p* < 0.001, *p < 0.05.

The molecular mechanisms of ZMPSTE24 and IFITM antiviral activity are not well characterised (Li et al., 2013, 2017; Fu et al., 2017). Findings from previous studies suggest that these proteins restrict the fusion of viruses by altering the fluidity or curvature of the host and viral membranes, through indirect alteration of the lipid composition of the endosomal membrane or through the association with membranous co-factors, such as ZMPSTE24 and IFITM interactions (Li et al., 2017; Wrensch et al., 2019). It has previously been demonstrated that the antiviral effect of IFITM2 and IFITM3 on infection by susceptible viruses such as IAV, can be attenuated in the presence of the amphiphilic antifungal drug amphotericin B (AmphoB; Lin et al., 2013; Wrensch et al., 2019). AmphoB intercalates into endosomal membranes and indirectly abrogates IFITM-mediated restriction through enhancement of membrane fluidity (Lin et al., 2013). We therefore used AmphoB treatment to analyse whether membrane modulation is required for ZMPSTE24 restriction and for the cooperative antiviral activity of ZMPSTE24 and IFITM proteins. To address this, we used CRISPR-Cas9 ZMPSTE24 KO A549 cells that overexpressed either ZMPSTE24 or IFITM3 individually or overexpressed both proteins in combination (Figure 5A). We infected these cells and CRISPR-Cas9 NT control cells with our panel of arenavirus GPpp namely LCMV, LASV, MOPV, LUJV, JUNV, MACV, and CHAPV. Notably, AmphoB had no effect on arenavirus GPpp infection in NT controls. We observed that AmphoB produced a broadly significant reversal of ZMPSTE24-mediated restriction of arenavirus GPpp infection, rendering these cells less sensitive to ZMPSTE24 inhibition (Figure 5A). Furthermore, AmphoB limited the cooperative restriction of ZMPSTE24 and IFITM3 in comparison to untreated cells (Figure 5A). These data imply that at the early stages of arenavirus infection, the modulation of cellular membrane integrity is critical for the antiviral activity of ZMPSTE24 and the observed restriction enhancement in the presence of IFITM3. To examine this during live virus infection, we infected these cells with MOPV at MOI 0.01 for 72 h and quantified levels of MOPV L and NP gene expression and infectious virus production. We found that AmphoB increased the sensitivity of MOPV to ZMSPTE24 alone and when expressed in combination with IFITM3 (Figure 5B). Similar to arenavirus GPpp infection (Figure 5A), expression of IFITM3 alone did not affect MOPV replication or production. Furthermore, we also observed no change in MOPV L and NP gene expression levels or infectious MOPV production upon AmphoB treatment in these cells.

Structural changes in host cell membranes that can affect, for example, membrane fluidity or surface tension have implications for a wide range of biological mechanisms including cell division, endocytosis and viral fusion (Lin et al., 2013; Torriani et al., 2019; Wrensch et al., 2019). Given that ZMPSTE24 and IFITMs act both independently and in synergy against a plethora of virus families, it is highly likely that they share a common mechanism that impacts on the host cellular environment. To investigate the impact of ZMPSTE24 on host membranes and indeed co-expression of the two proteins, we assessed the rate of cellular internalisation of the non-toxic membrane probe FM™2-10 in A549 cells overexpressing ZMPSTE24, IFITM3 or ZMPSTE24 and IFITM3 in combination. FM™2-10 reversibly binds to the outer leaflet of the cell membrane and upon endocytosis localises to the membrane of the endocytic vesicle (Richards et al., 2000). The fluorescence emission of FM™2-10 increases with membrane incorporation, thus we measured the changes in FM™2-10 fluorescence intensity over time by flow cytometry to determine the kinetics of membrane internalisation. In contrast to control cells, the rate and intensity of FM™2-10 probe incorporation, characteristic of membrane endocytosis, was reduced in the presence of IFITM3 or ZMPSTE24 and this reduction was significantly enhanced upon co-expression of the two restriction factors (Figure 5C). These findings strengthen the argument that ZMPSTE24 and IFITMs cause changes in membrane structure and dynamics that likely impact on the efficiency of virus fusion.

Thus, ZMPSTE24-mediated restriction activity is involved in the early stages of the innate immune response to arenavirus infection and IFITMs are able to enhance the effects on membrane fluidity and thus inhibition of virus infection.

## Discussion

The entry and fusion of viruses into susceptible host cells represents a fundamental step of viral pathogenesis and is a central factor in disease outcome. Investigations into the molecular and cellular mechanisms of arenavirus infection have unravelled complex details surrounding receptor switching, regulation of virus endocytosis and conformational rearrangements within the GP structure that induce membrane fusion (Li et al., 2016; Hulseberg et al., 2018). However, there is still limited knowledge regarding the range of antiviral proteins that limit arenavirus entry and how their activity may be modulated by virus-specific proteins.

In this study, we identified ZMPSTE24 as an intrinsic restriction factor against arenavirus entry and replication. The antiviral impact of ZMPSTE24 on arenavirus infection was shown by demonstrating that arenavirus GPpp infection and MOPV replication are enhanced in cells with depleted ZMPSTE24 and that ectopic expression of ZMPSTE24 caused a reduction in arenavirus GPpp and MOPV infection. Recent studies have indicated a number of enveloped viruses that traffic through the cellular endosomal compartment during entry are restricted by ZMPSTE24 (Fu et al., 2017). Our findings now expand this list to include arenaviruses. The breadth of viruses impacted by ZMPSTE24 activity suggests a universal antiviral mechanism that occurs prior to virus fusion. Our data suggests that disrupting the protease activity of ZMPSTE24 does not alter the sensitivity of arenaviruses to restriction, further implying a generalised mechanism of restriction that may involve modifying host cell membrane properties to inhibit fusion pore formation. This mechanism which impairs endosomal viral membrane fusion is likely facilitated by the IFITM proteins as co-factors of ZMPSTE24 activity.

The localisation of IFITM proteins is an important determinant of the breadth of viruses that they restrict. IFITM1 is found predominantly at the plasma membrane whilst IFITMs 2 and 3 localise to endosomal compartments (Foster et al., 2016). A recent study by Suddala et al. (2019) indicated that IFITM3 restricts through a proximity-based mechanism and that LASV may escape restriction by IFITM3 by entering cells through a distinct endosomal pathway lacking IFITM3 expression. Given that previous studies have showed that ZMPSTE24 is required for the antiviral activity of IFITMs, we explored a possible cooperative role of ZMPSTE24 and IFITMs against arenavirus infection. Our data demonstrate that endogenous ZMPSTE24 co-localises with all three IFITM proteins when they are ectopically expressed in A549 cells. Using immunoprecipitation and complementation assays, we also showed that IFITM proteins interact with ZMPSTE24.

In our present study, we provide strong evidence for the biological significance of the ZMPSTE24—IFITM interaction demonstrating that engagement with IFITM proteins enhances the sensitivity of arenaviruses to ZMPSTE24-mediated restriction. Specifically, we show that stable ectopic expression of ZMPSTE24 with IFITM3 significantly inhibited arenavirus entry and replication. In addition, we found that in contrast to cells singly overexpressing IFITM3, ectopic co-expression of IFITM3 with ZMPSTE24 in A549 cells led to the redistribution of IFITM3 to distinct endosomal compartments that were positive for ZMPSTE24. We therefore propose the redistribution of IFITM3 to a ZMPSTE24-positive pathway along which arenaviruses enter and fuse, induces an enhanced modification of cellular membranes that impairs virus fusion. Supporting this hypothesis, we provide evidence that the absence of IFITMs in A549 cells expressing ZMPSTE24 leads to a reduction in the sensitivity of arenavirus GPpp to ZMPSTE24-mediated restriction. We may therefore hypothesise that ZMPSTE24 can modulate intracellular trafficking of IFITM co-factors to an early endosomal localisation that increases the susceptibility of IFITM-resistant viruses like arenaviruses.

We aimed to address the mechanism of ZMPSTE24 restriction and of the observed cooperative activity using AmphoB treatment which disrupts IFITM function and by assessing the incorporation of a membrane-sensitive probe, FM™2-10. Pre-treatment with AmphoB rescued the entry and fusion of arenavirus GPpp and live MOPV infection in cells expressing either ZMPSTE24 alone or co-expressing ZMPSTE24 and IFITM3. These findings are consistent with the notion that ZMPSTE24 may exert its inhibitory effect by modulating the curvature and increasing the rigidity of endosomal membranes, much like the IFITM proteins. ZMPSTE24 decreased the rate and intensity of FM™2-10 incorporation into cellular membranes and this was further abrogated in the presence of IFITM3. Given the effects that these proteins likely have on membrane fluidity, the decreased incorporation and thus associated reduced rate of endocytosis may be an indirect effect of changes in lipid composition and distribution of membrane components. It also provides evidence that both ZMPSTE24 and IFITM3 exert their antiviral function by increasing membrane order and rigidity, a mechanism consistent with that proposed by previous studies on IFITM3 alone (Li et al., 2013; Lin et al., 2013).

In summary, we highlight a previously unexplored restriction strategy that contributes to our understanding of arenavirus entry mechanisms. It provides further insight into the activities of ZMPSTE24 and IFITMs and provides the opportunity to target and augment this restriction mechanism for antiviral development. Defining the critical interaction sites between ZMPSTE24 and IFITM proteins and understanding the role of arenaviral proteins in abrogating restriction is of importance.

## Data Availability Statement

The raw data supporting the conclusions of this article will be made available by the authors, without undue reservation.

## Author Contributions

TF conceived the project and obtained the funding. RS-M and TF designed and performed the experiments. Both authors analysed the data and wrote the manuscript.

## Conflict of Interest

The authors declare that the research was conducted in the absence of any commercial or financial relationships that could be construed as a potential conflict of interest.

## Publisher’s Note

All claims expressed in this article are solely those of the authors and do not necessarily represent those of their affiliated organizations, or those of the publisher, the editors and the reviewers. Any product that may be evaluated in this article, or claim that may be made by its manufacturer, is not guaranteed or endorsed by the publisher.
