# Supplementary material for: Inhibition of Arenavirus Entry and Replication by the Cell-Intrinsic Restriction Factor ZMPSTE24 Is Enhanced by IFITM Antiviral Activity
Source: Front Microbiol. 2022 Feb 18;13:840885. doi: 10.3389/fmicb.2022.840885 (PMC8915953; doi:10.3389/fmicb.2022.840885)
Supplement: Supplementary file 1 [file Data_Sheet_1.PDF]

Supplementary Material

Supplementary Figures

Supplementary Figure 1. ZMPSTE24 inhibits arenavirus infection.

(A) Representative gating strategy for flow cytometry analysis of arenavirus-GP pseudotyped virus infection. The selected cell population was gated for GFP positive cells (FITC channel) using uninfected cells as a negative control. **(B)** Representative gating strategy for flow cytometry analysis of BlaM-Vpr assay for viral fusion. A549 cells stably overexpressing ZMPSTE24 or empty vector control cells were exposed for 3h with pseudoparticles containing BlaM-Vpr and expressing the GP proteins of LCMV, LASV or MOPV, prior to loading with CCF2-AM. The cells were assayed for cleavage of CCF2-AM by measuring the conversion of uncleaved CCF2-AM (fluorescence emission from 520 nm) to cleaved CCF2-AM (fluorescence emission 447 nm) using flow cytometry. Fusion of the BlaM-Vpr pseudoparticles was inhibited by bafilomycin A1 (BafA1). **(C)** Representative MOPV plaque images of supernatant samples from A549 CRISPR-Cas9 non-targeting (NT) control, ZMPSTE24 knockout (KO) or ZMPSTE24 KO cells overexpressing ZMPSTE24. 10-fold dilutions of samples indicated. **(D)** A549 cells stably overexpressing ZMPSTE24 with the H335A mutation were infected with LCMVpp or LASVpp and infectivity was measured as %GFP positive cells by flow cytometry after 48h. **(E)** A549 CRISPR-Cas9 NT control or ZMPSTE24 KO was confirmed by western blot (inset) and cells were infected with arenavirus GPpp for 48h. infectivity was measured as %GFP positive cells by flow cytometry. *$p<0.05$.

Supplementary Figure 2. IFITMs contribute to ZMPSTE24 restriction activity.

(**A**) Representative MOPV plaque images of 10-fold serially diluted supernatant samples from A549 cells treated with or without type 1 interferon (IFN1) at 72h post infection. **(B)** Representative gating strategy for flow cytometry analysis of LCMV-GP and LASV-GP pseudotyped virus infection of A549 CRISPR-Cas9 non-targeting (NT) control, ZMPSTE24 knockout (KO) or ZMPSTE24 KO cells overexpressing ZMPSTE24, IFITM3 or both. The selected cell population was gated for GFP positive cells (FITC channel) using uninfected cells as a negative control. **(C)** Representative gating strategy for flow cytometry analysis of LCMV-GP and LASV-GP pseudotyped virus infection of A549 CRISPR-Cas9 non-targeting (NT) control, NT control cells overexpressing ZMPSTE24, IFITM knockout (KO) or IFITM KO cells overexpressing ZMPSTE24. The selected cell population was gated for GFP positive cells (FITC channel) using uninfected cells as a negative control.
