# Supplementary figures and images for: Inhibition of Arenavirus Entry and Replication by the Cell-Intrinsic Restriction Factor ZMPSTE24 Is Enhanced by IFITM Antiviral Activity
Source: Front Microbiol. 2022 Feb 18;13:840885. doi: 10.3389/fmicb.2022.840885 (PMC8915953; doi:10.3389/fmicb.2022.840885)

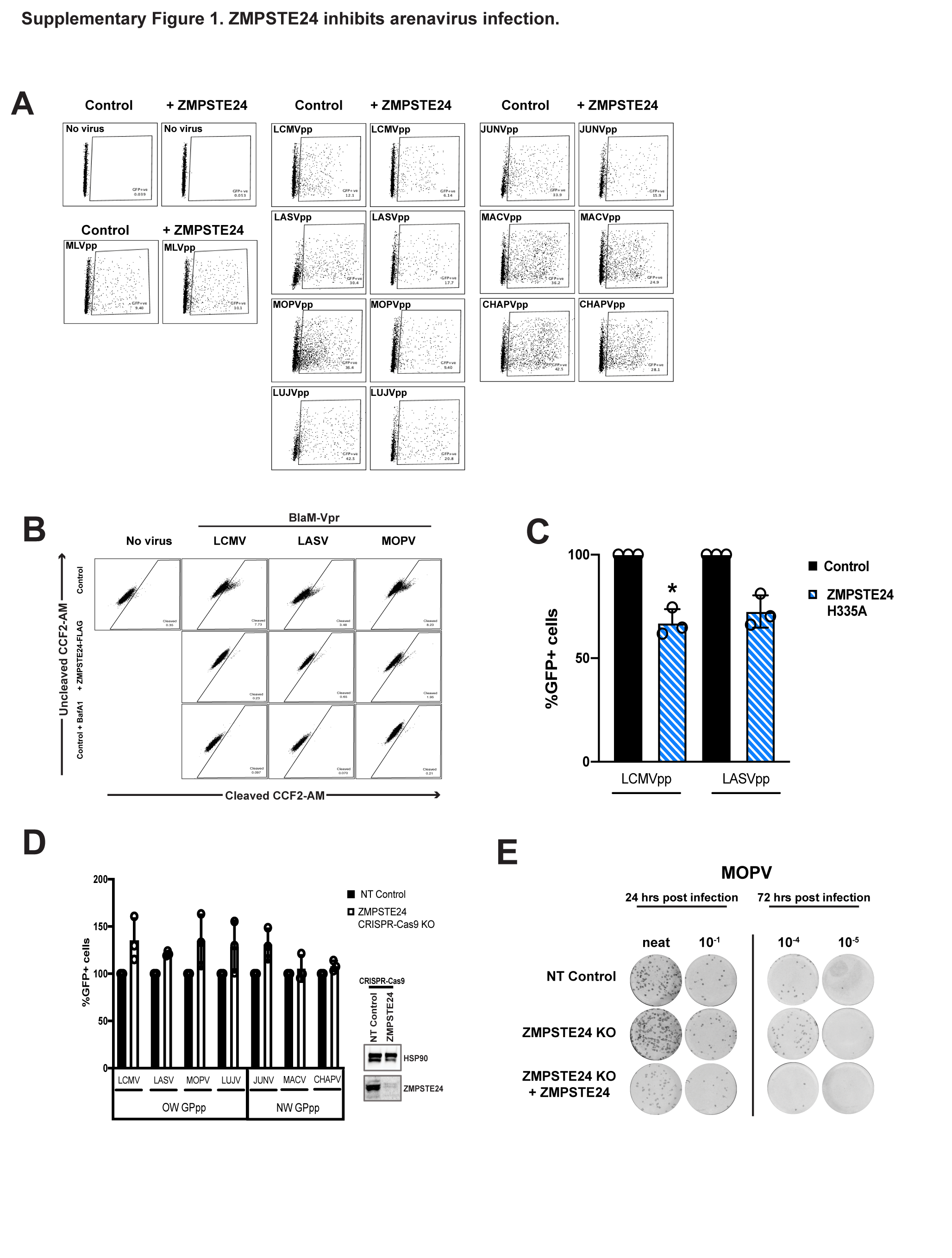

Supplement: Supplementary file 2 [file Image_1.TIF]

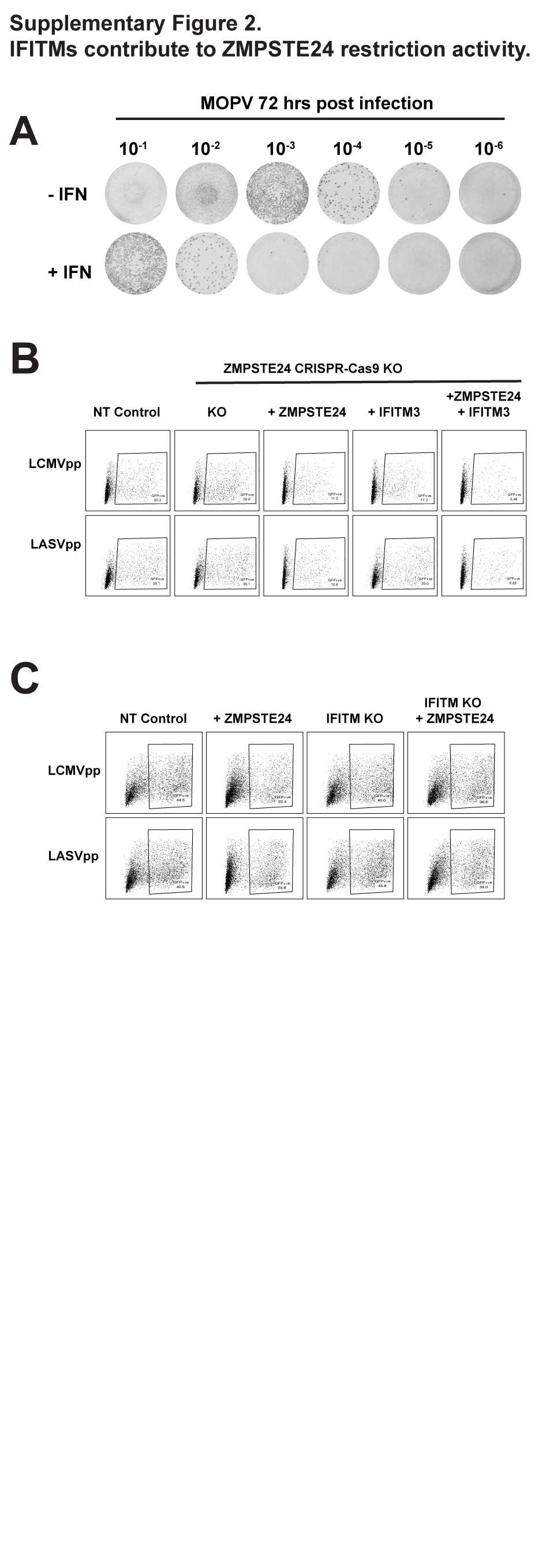

Supplement: Supplementary file 3 [file Image_2.TIF]
